# The Value of Total Body Photography for the Early Detection of Melanoma: A Systematic Review

**DOI:** 10.3390/ijerph18041726

**Published:** 2021-02-10

**Authors:** Annkathrin Hornung, Theresa Steeb, Anja Wessely, Titus J. Brinker, Thomas Breakell, Michael Erdmann, Carola Berking, Markus V. Heppt

**Affiliations:** 1 Department of Dermatology, Universitätsklinikum Erlangen, Friedrich-Alexander-University Erlangen-Nürnberg (FAU), 91054 Erlangen, Germany; Annkathrin.Hornung@uk-erlangen.de (A.H.); Theresa.Steeb@uk-erlangen.de (T.S.); Anja.Wessely@uk-erlangen.de (A.W.); breakell.tom@gmail.com (T.B.); michael.erdmann@uk-erlangen.de (M.E.); carola.berking@uk-erlangen.de (C.B.); 2 Comprehensive Cancer Center Erlangen—European Metropolitan Region of Nürnberg, 91054 Erlangen, Germany; 3 Digital Biomarkers for Oncology Group, National Center for Tumor Diseases (NCT), German Cancer Research Center (DKFZ), 69120 Heidelberg, Germany; titus.brinker@nct-heidelberg.de

**Keywords:** artificial intelligence, early detection, melanoma, photography, prevention, skin cancer, total body photographic cutaneous surveillance

## Abstract

Early detection of melanoma is critical to reduce the mortality and morbidity rates of this tumor. Total body photography (TBP) may aid in the early detection of melanoma. To summarize the current evidence on TBP for the early detection of melanoma, we performed a systematic literature search in Medline, Embase, and the Cochrane Central Register of Controlled Trials (CENTRAL) for eligible records up to 6th August 2020. Outcomes of interest included melanoma incidence, incisional and excisional biopsy rates, as well as the Breslow’s index of detected tumors. Results from individual studies were described qualitatively. The risks of bias and applicability of the included studies was assessed using the QUADAS-2 checklist. In total, 14 studies published between 1997 and 2020 with an overall sample size of *n* = 12082 (range 100–4692) were included in the qualitative analysis. Individuals undergoing TBP showed a trend towards a lower Breslow’s thickness and a higher proportion of in situ melanomas compared to those without TBP. The number needed to excise one melanoma varied from 3:1 to 14.3:1 and was better for lesions that arose de novo than for tracked ones. The included studies were judged to be of unclear methodological concern with specific deficiencies in the domains “flow and timing” and “reference standard”. The use of TBP can improve the early detection of melanoma in high-risk populations. Future studies are warranted to reduce the heterogeneity of phenotypic risk factor definition and the technical implementation of TBP. Artificial intelligence-assisted analysis of images derived from 3-D TBP systems and digital dermoscopy may further improve the early detection of melanoma.

## 1. Introduction

The incidence of cutaneous melanoma continues to rise steadily in Western countries with Caucasian populations each year [1,2]. Surgical treatment is usually curative following early detection of the disease [3,4]. However, melanoma quickly becomes life-threatening once it metastasizes [3], although considerable progress has been made following novel therapeutic interventions including BRAF and MEK inhibitors and immunotherapies [5,6]. Thus, early detection of cutaneous lesions remains of paramount importance to significantly reduce the morbidity and mortality caused by melanoma, as thinner primary tumors are associated with an improved survival rate [7,8,9]. Hence, strategies to improve early detection are critical in patients at high risk for melanoma.

In Germany, the worldwide unique national skin cancer screening program was implemented in 2008. It involves a voluntary, standardized full-body examination by dermatologists or general practitioners specifically trained for this purpose. The costs are reimbursed by all German statutory health insurance companies every two years for members older than 35 years, while some health insurance companies also cover the costs for younger members [10]. Instead of mass screening of an entire population, other approaches aim to selectively identify and surveil individuals at the highest risk for melanoma [11,12]. One of the most promising approaches involves automated imaging of patients or total body photography (TBP), allowing objective documentation of all existing lesions and monitoring changes over time. TBP uses clinical photography of the patients’ skin surface to provide a photographic record. This can be achieved by either 2-D or 3-D TBP. In 2-D TBP, common 2-D images of the skin are taken and composed to a body map while a 3-D representation of the patient’s surface is created in 3-D TBP, partly even linked with dermoscopy images. Thus, 3-D TBP may particularly facilitate the localization of lesions for follow-up [13]. Both techniques may aid in the assistance of the early detection of melanoma as they improve the diagnostic accuracy of melanoma in high-risk patients [14,15,16,17]. TBP has the advantage of monitoring the patient’s entire skin surface, rather than only a subset of individual lesions. Furthermore, TBP can be combined with sequential digital dermoscopy imaging (SDDI) as this “two-step approach” might provide more intensive surveillance in high-risk patients to assist with an early melanoma diagnosis.

However, until now, the value of TBP in the early detection of melanoma has not been systematically investigated. Here we performed a systematic review to summarize the current publication landscape of TBP in the prevention of melanoma. Our results will assist dermatologists as well as patients in choosing an appropriate and individual screening and surveillance strategy.

## 2. Materials and Methods

This review was conducted in accordance with the Preferred Reporting Items for Systematic Reviews and Meta-Analyses (PRISMA) [18], its extension for diagnostic test accuracy [19,20], and the Cochrane Handbook for Systematic Reviews [21].

### 2.1. Eligibility Criteria

We included studies investigating patients of any age with common melanocytic nevi (moles), congenital nevi, or dysplastic/atypical nevi undergoing 2-D TBP or 3-D TBP to detect melanoma early as part of screening for surveillance. Combination with other diagnostic measures, such as physical examination, self-examination, or SDDI was allowed but not obligatory for inclusion. In addition, studies investigating only TBP for keratinocyte cancer were excluded from the analysis.

Regarding the study design, we included randomized controlled trials (RCTs), clinically controlled trials (CCTs), non-controlled prospective trials, prospective observational studies, case-control studies as well as retrospective studies. Narrative reviews and case reports or series were excluded. No language restrictions were set. In RCTs and controlled observational studies, usual care served as control. If no control group was specifically included, data from external control groups or the general population served as control.

### 2.2. Types of Outcome Measures

The outcomes of interest were all related to the early detection of melanoma. Thus, the incidence of melanoma, the number of excisions and biopsies, the number of changed or new nevi, as well as the benign-to-malignant ratio of excised lesions were defined as outcomes of interest. Additionally, the mean or median vertical invasion depth measured in mm (Breslow’s index) of detected melanomas was considered as an outcome. As we expected heterogeneously reported outcomes, we extracted the data as reported in the publication and did not perform any re-calculations.

### 2.3. Search Methods for Identification of Studies

We searched the electronic databases Medline, Embase (both via Ovid), and the Cochrane library CENTRAL until 6 August 2020 to identify all possibly relevant records (Table 1). For ongoing trials and completed trials without data publication, principal investigators or trial sponsors were contacted to obtain preliminary or unpublished data. Moreover, reference lists of included records and evidence- and consensus-based guidelines on the diagnosis of melanoma were screened.

### 2.4. Selection of Studies

Three authors (T.S., A.H., M.V.H.) independently screened titles and abstracts for eligibility that were identified in the electronic database searches. For records that were considered relevant according to title and abstract screening, full-text articles were obtained, and inclusion and exclusion criteria were applied by the same three authors. Whenever discrepancies arose, a resolution was achieved by discussion with another independent author (C.B., A.W., T.J.B., T.B., M.E.).

### 2.5. Data Extraction and Management

Information for each included study regarding study design, baseline characteristics of the included population, risk factors for melanoma, imaging technique, and intervals of TBP, follow-up strategy, main outcomes, and study limitations were collected and summarized independently by two authors (A.H., M.V.H.). Data were extracted to an internally piloted data extraction spreadsheet using Microsoft Excel 2010. If multiple reports of a primary study were identified, all available data were extracted. According to the principles of evidence-based medicine, we also decided to contact study authors for clarification in case of inconsistencies in reporting or overlapping study populations were identified. However, this was not the case for any of the identified records. The baseline characteristics and the outcomes of interest of each study were described qualitatively within the text, as a quantitative synthesis was not feasible due to the high heterogeneity of included studies.

### 2.6. Assessment of Methodological Quality

The risks of bias and applicability of the included studies were assessed by two reviewers (A.H., T.S.) independently using a modified version of the QUADAS-2 checklist [22]. QUADAS-2 consists of the four key domains “patient selection”, “index test”, “reference standard”, and “flow and timing”. Each domain was assessed by the two raters in terms of risk of bias. The first three domains were additionally rated in terms of concerns regarding applicability. Any disagreement was resolved by consensus with another review author (M.V.H.).

## 3. Results

### 3.1. Study Identification

**Figure 1:**
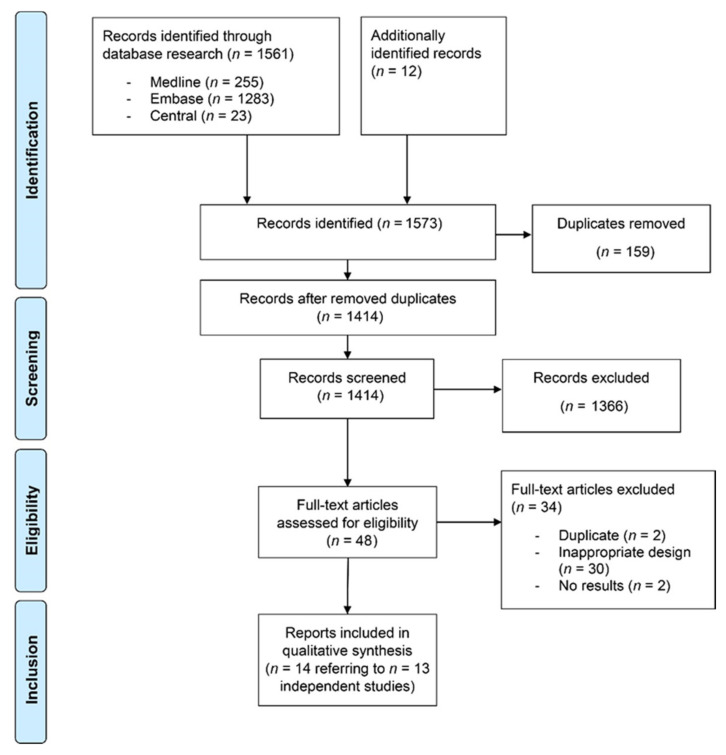
Flow chart of the literature identification process according to the PRISMA guidelines.

We identified 1573 references through database searching and cross-referencing in guidelines. After removal of duplicates and title-abstract screening, 48 records underwent full-text review. Thirty records were dismissed as the study design did not meet the predefined eligibility criteria or as the record did not refer to a study. In addition, two further duplicates were identified, and two references did not present any results. Hence, 14 published reports referring to 13 independent studies published between 1997 and 2020 with an overall sample size of *n* = 12082 (range 100–4692) were included in qualitative analysis (Figure 1).

### 3.2. Study Characteristics

Seven studies were conducted in the USA [23,24,25,26,27,28,29], and three in Australia [30,31,32]. One study was performed in Europe [33,34], one in New Zealand [35], and one in Israel [36] (Table 2). All included studies investigated 2-D TBP while none investigated 3-D TBP. However, four studies did not describe the methods of imaging in detail [24,25,27,29], while in eight studies TBP was performed by using standardized poses or views with a systematic series of photos [26,28,30,31,32,33,34,35,36]. In the study performed by Drugge et al., a semi-automated TBP system was deployed which captured images simultaneously using 25 cameras [23,29]. While in six studies TBP was performed with serial imaging [23,29,31,33,34,35,36], in four studies only baseline TBP was performed [26,28,30,32]. In addition, ten studies also implemented dermoscopy [23,26,28,29,30,31,32,33,34,35,36]. SDDI was only conducted in four studies [31,33,34,35,36]. In most studies, 12–27 macroscopic images were acquired with conventional cameras to capture the entire skin surface in 2-D [26,30,31,32,36].

The selection and inclusion criteria of the screened populations were highly heterogeneous. Risk factors for inclusion differed considerably between the studies. In four publications, no further risk factors were specified for study participation [23,25,27,28] (Table 2).

### 3.3. Outcomes

#### 3.3.1. Prospective Studies

Banky et al. performed a cohort study of 309 patients at high risk for melanoma who underwent baseline TBP and were followed up at three-, six-, or twelve-month intervals [32]. Eighteen melanomas were detected. The overall incidence of melanoma was 19 per 1000 person-years. The benign-to-malignant ratio of lesion biopsies was almost 3:1.

**Figure 2:**
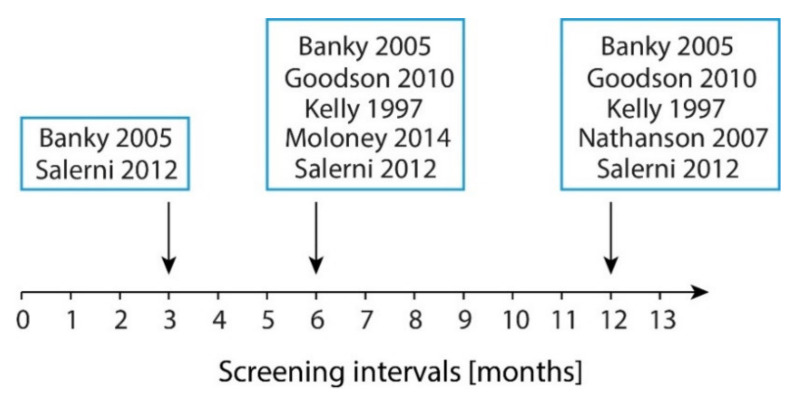
Imaging intervals of the prospective trials. Screening intervals according to intervals in months.

In the prospective cohort study reported by Goodson et al., TBP was performed at baseline and patients were clinically followed-up at six- and twelve-month intervals (Figure 2). Outcomes of interest were the biopsy rate, the efficiency of melanoma detection, and melanoma origin (de novo or nevus-derived). In total, 548 biopsies were performed in 1076 patients (273 during the initial visit and 275 on follow-up) [26]; 61% of the 275 biopsies at follow-up were performed due to photographic evaluation. At follow-up, 12 melanomas were detected, 5 were invasive. Five of them presented as changing and two as new lesions.

Kelly et al. assessed 278 patients with dysplastic melanocytic nevi in a prospective cohort study [30]. Patients were followed up for a mean of 42 months, 210 biopsies were obtained. Overall, 20 new melanomas (*n* = 12 invasive, *n* = 8 in situ) were detected in 16 patients. The age-adjusted incidence was calculated to 1835/100,000 person-years. Notably, 11 melanomas were detected due to comparison with baseline photographs.

Moloney et al. performed a prospective observational study in Australia to examine the effect of regular full body examination in combination with dermoscopy and TBP [31]. SDDI was performed for ambiguous lesions. In 311 patients, 75 primary melanomas were detected, 14 of them at baseline visit; 38% were detected using TBP, and 39% with SDDI. Of the 770 excised lesions, 441 were melanocytic, including 82 melanomas, representing a 4.4:1 benign-to-malignant melanoma ratio. The overall rate of melanomas identified during follow-up per study year was 0.08.

Nathansohn et al. established a pigmented lesions clinic based on a digital photography studio with TBP and dermoscopy. In the first 20 months of the prospective study, 895 patients were examined, 206 of them had follow-up visits [36]; 236 suspicious lesions were excised with 7 emerging as melanomas.

Taken together in all six prospective studies 7 to 98 melanomas were detected. Two of these studies indicated that up to 11 out of 20 melanomas were detected due to TBP. Three studies specified the number needed to excise, ranging from 3:1 to 11.9:1. Altogether the number of biopsies ranged from 210 to 1152 biopsies (Table 3 and Table 4).

#### 3.3.2. Retrospective Studies and Chart Reviews

In the retrospective study by Drugge et al., 4692 patients were analyzed regarding patient characteristics, self-reported melanoma risk factors, and the usage of TBP [23]; 2473 of the patients were scanned at least once with TBP, 268 biopsies were obtained in 218 patients. At least one malignant lesion was identified in 65 of the 218 patients (30%). The number needed to excise one melanoma for all 268 lesions was 3:1. The ratio of melanoma in situ to invasive melanoma was 1.56:1. Using data from the same cohort, the frequency of TBP and the invasion depth of identified melanoma were correlated in 218 patients. In total, 225 lesions were biopsied, 67 (29.8%) of which were malignant, including 44 in situ and 23 invasive melanomas [29]. The minimum interval between baseline and a follow-up TBP session to identify a new invasive melanoma was 1.29 years.

Feit et al. reported the results of a retrospective study including 576 patients [28]; 93 suspicious lesions were excised of which 77 were melanocytic. Photographic assistance identified 12 patients with 27 histologically confirmed melanomas (*n* = 21 in situ; *n* = 6 invasive); 74% of the melanomas (*n* = 27) were biopsied due to changes from baseline while 19% were biopsied because they newly emerged.

The retrospective registry review of two cohorts by Rademaker et al. investigated demographic and histological data from 100 melanomas diagnosed through self-referred TBP and SDDI service compared to those diagnosed through traditional methods [35]. Fifty-two invasive and 48 in situ melanomas were identified; 48 were diagnosed at the first visit and the remaining by serial digital imaging.

Risser et al. compared biopsy numbers in 946 patients with multiple atypical nevi in their first year of care who received either total body skin examination alone or in combination with TBP [24]. The mean number of performed biopsies was similar in both groups (skin examination only vs. skin examination plus TBP: 0.82 vs. 0.8). Three melanomas were diagnosed in 19 patients who did not receive TBP, whereas no melanoma was diagnosed in 16 patients receiving TBP.

Salerni et al. analyzed the use of TBP and dermoscopy in 618 high-risk melanoma patients. A total of 1152 lesions in 407 patients were excised during the surveillance period, corresponding to a mean of 2.83 lesions per patient during a 10-year follow-up period (global excision rate 1.86 per patient in 618 patients) [33,34]; 98 melanomas were detected in 78 patients (*n* = 53 in situ), comprising 8.5% of excised lesions. Sixty melanomas were tracked lesions (benign-to-malignant ratio 11.9:1) while 38 melanomas newly emerged (benign-to-malignant ratio 8.8:1).

In the retrospective study performed by Strunck et al., 121 melanomas were diagnosed among 1,955 patients receiving TBP (51 melanoma in situ, 70 invasive melanomas) [27]. Of the 121 biopsies, 66 (54.5%) were performed due to suspicious photographs. Twelve of 121 (9.9%) melanomas newly emerged. TBP identified 11 of them (91.6%). In contrast, TBP identified 54 of 65 (83%) melanomas which were tracked as changing lesions.

Truong et al. reviewed the records of 926 patients who received TBP and had two or more follow-up visits over two years or longer [25]. Patients with TBP received 1.56 biopsies on average. In 1419 biopsies 93 primary melanomas were detected (49% melanoma in situ), corresponding to a combined benign-to-malignant ratio of 14.3:1.

Altogether in seven retrospective studies or chart reviews the number of invasive melanomas were reported in six studies and ranged from 0 to 70. The number needed to excise was specified in two studies and ranged from 3.1:1 to 14.3:1. The number of biopsies in patients with TBP ranged from 53 to 921 biopsies (Table 3 and Table 4).

### 3.4. Breslow Index of Invasive Melanoma

The reported depth of detected melanoma varied across all studies (Table 3 and Table 4). However, individuals undergoing TBP showed a trend towards a lower Breslow’s thickness. Patients diagnosed by TBP and SDDI screening had thinner melanomas compared to the registry data in the study by Rademaker et al. (69% vs. 52% <0.75 mm; 1.9% vs. 10.8% >3 mm) [35]. Additionally, a lower index was associated with having one or more follow-up visits (median 0.83 vs. 0.33 mm) and photographic review (median 0.31 vs. 0.48 mm) in the study by Strunck et al. [27]. The median depth was 0.31 mm (range 0.11–1.5) if TBP could be used compared to 0.48 mm (0.10–3.3) without consulting of TBP. Additionally, depths of tumors detected with TBP ranged from 0.11 to 2.1 mm vs. 0.2 to 3.0 mm for those detected without TBP in the study by Truong et al. [25].

### 3.5. Methodological Quality of Included Studies

**Figure 3:**
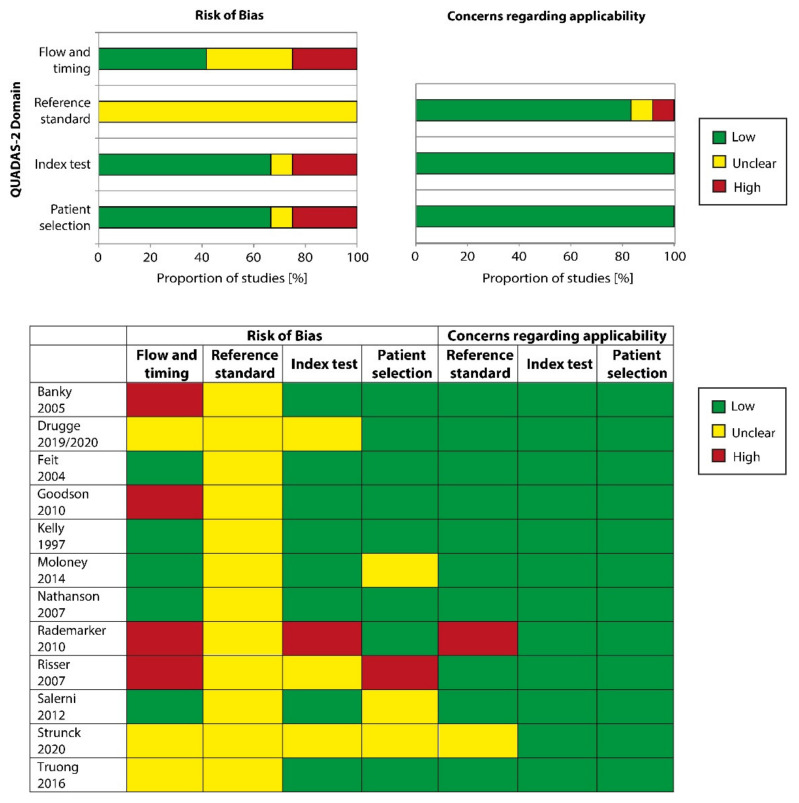
Risk of bias and applicability concerns graph: review authors’ judgments about each domain presented as percentages across included studies. Risk of bias and applicability concerns summary: review authors’ judgments about each domain for each included study.

Overall, the included studies were judged to be of unclear methodological concern (Figure 3). At least half of the studies were at high or unclear risk of bias for “flow and timing” and all studies were at high risk for bias regarding the “reference standard”, mostly since it was unclear whether the reference standard results had been interpreted without knowledge of the results of the index test. Approximately one-third of the studies showed an increased risk in the domain “patient selection” due to unclear sampling of participants. An additional third of the studies had an increased risk in the domain “index test” as it remained unclear if the index test results had been interpreted without knowledge of the results of the reference standard. However, the applicability of study findings was of low concern for the majority of studies in the domains “index test” and “patient selection”, while the applicability was of unclear or high concern for the domain “reference standard” in less than 20% of the studies.

## 4. Discussion

In this study, we aimed to investigate the value of TBP in the early detection of melanoma. Overall, we have identified 14 studies with an overall sample size of *n* = 12,082 highlighting that individuals undergoing TBP showed a trend towards a lower Breslow’s thickness and a higher proportion of in situ melanomas compared to those without TBP. In addition, our results show that the use of TBP can improve the early detection of melanoma in high-risk populations.

For the early detection and thus secondary prevention of melanoma, there are two distinct strategies to select the target population. Firstly, untargeted mass screening programs can be performed (population-based screening) regardless of individual risk profiles. However, this procedure is only carried out in a few countries such as Germany and is costly due to the high number of screened individuals [37]. In Australia, the number needed to screen to save one life from melanoma was calculated to 25,000 individuals [38]. Hence, the effect in terms of significantly reduced mortality is controversial and considerable resources are needed for a nationwide implementation of such a screening program. Secondly, screening efforts can be restricted to populations at increased risk of developing melanoma. This procedure is more targeted, and the expected effects are larger because the prevalence of the screened population is significantly higher compared with the normal population. The latter procedure was used in the studies reviewed here, yet the definition of risk factors was fairly heterogeneous in keeping with previous reviews on risk prediction models [39]. In most studies, the presence of >4 to 5 clinically atypical nevi or a large number (>100) of common nevi was required for investigation by TBP. A positive personal or family history of melanoma was also among the inclusion criteria in some studies while confirmed genetic susceptibility was reported only for two studies. From these data, we conclude that the screened populations had a high to very high risk for melanoma. However, four studies did not specify the risk profiles, underlining that there is currently no consensus on the best risk assessment [40,41]. For the conduct of RCTs, risk prediction tools should be harmonized and include a weighting of individual factors to enable cross-population comparisons of distinct regions and ethnicities.

The technical implementation and timing intervals of TBP were similarly heterogeneous as the definition of clinical risk factors. In most studies, 12–30 macroscopic images were acquired with conventional cameras to capture the entire skin surface in two dimensions (2-D TBP). As a limitation, multiple imaging systems were applied with distinct resolutions. Furthermore, the study protocols only referred to standard poses and often did not specify exactly how the images were acquired. Specific anatomic regions such as intertriginous areas, genitals, retroauricular area, or the flexor sides of the extremities may not always be adequately captured with 2-D TBP. Here, the use of 3-D TBP systems in defined postures can help to standardize the image acquisition as they generate a digital 3-D avatar of the imaged person that can be used to analyze and prospectively follow all nevi of interest [13,37,42]. Currently, an RCT is ongoing in Australia evaluating the efficacy of 3-D TBP combined with SDDI compared with regular skin examinations which may or may not use 2-D TBP with SDDI. The main outcomes to be prospectively assessed in this study are the number of incisional and excisional biopsies and the Breslow depth of detected melanomas [37]. The number of in situ tumors also represents a valid indicator for the early detection of melanoma.

The results of our study clearly show that 2-D TBP identified a higher proportion of in situ tumors and melanomas with a lower average Breslow thickness compared with the comparison groups without 2-D TBP. The number needed to excise one melanoma varied widely, ranging from 3:1 to 14.3:1. This ratio was better for lesions that arose de novo than for tracked ones. We conclude that the latter need to be assessed with additional methods such as dermoscopy and that the added value of TBP is highest for lesions that arose newly during the surveillance period.

Artificial intelligence (AI) was not used in any of the included studies to evaluate the captured images. The image analysis was performed by the respective physicians in all reports. Several landmark studies have recently shown that AI performed on par with dermatologists in the distinction of nevi from melanoma based on dermoscopic images [43,44,45]. Similarly, future studies are warranted to investigate whether the analysis of 3-D TBP images using AI is also feasible. AI ultimately should be integrated into 3-D TBP systems to support image analysis, pattern recognition, and detect changes of nevi in sequential examinations. Furthermore, the 3-D TBP images should also be equipped with interfaces to SDDI, which in turn could also be analyzed using AI to make screening efforts more accurate while maintaining cost efficiency.

Overall, our study shows that the use of 2-D TBP can improve the early detection of melanoma in high-risk populations. However, the definition of phenotypic risk factors, technical performance of TBP, and design of the included studies were highly heterogeneous. In the future, harmonized risk prediction tools should be developed to compare distinct populations. 3-D TBP with the generation of digital avatars is a game-changing technology and will enable standardized and gapless image acquisition.

## Figures and Tables

**Table 1:** Detailed Strategy of the Literature Identification Process.

Ovid MEDLINE(R) and Epub Ahead of Print, In-Process & Other Non-Indexed Citations, Daily and Versions(R) 1946 to 04 August 2020 *n* = 255
exp Imaging, Three-Dimensional/ or vectra.mp. ^1^ whole Body photography.mp. 3D total Body photography.mp. total-Body 3D photography.mp. complete Body photography.mp. Body photography.mp. exp Skin Neoplasms/ or exp Melanoma/ or cutaneous melanoma.mp. 1 or 2 or 3 or 4 or 5 or 6 7 and 8
**Embase 1974 to 2020 Week 31, *n* = 1283**
vectra.mp. Three-Dimensional Imaging.mp. or exp three-dimensional imaging/ or exp image processing/ whole Body photography.mp. exp imaging system/ or 3D total Body photography.mp. total-Body 3D photography.mp. complete Body photography.mp. Body photography.mp exp skin cancer/ or cutaneous melanoma.mp. or exp melanoma/ or exp cutaneous melanoma/ 1 or 2 or 3 or 4 or 5 or 6 or 7 8 and 9
**Cochrane library, *n* = 23 trials**
melanoma OR cutaneous melanoma Vectra Three-dimensional imaging 3D total Body photography total-Body 3D photography complete Body photography Body photography #2 OR #3 OR #4 OR #5 OR #6 OR #7 #1 AND #8

^1^ “mp” refers to an ovid syntax term standing for multi-purpose. In general, an MP search searches the title, original title, abstract, subject heading, name of the substance, and registry word fields.

**Table 2:** Baseline Characteristics of the Included Studies.

Study	Design	Population	Risk Factors for Inclusion	Imaging Technique	Baseline vs. Serial TBP
Prospective Trials					
Banky 2005	prospective single cohort	>16 years median age 38 years (range 16–74) *n* = 309 (female *n* = 168, male *n* = 141) Australia	>4 dysplastic nevi >100 nevi positive family history of melanoma positive personal history of melanoma	14 baseline photographs 35 mm single-lens reflex camera dermoscopy allowed, no SDDI	baseline TBP
Goodson 2010	prospective single cohort	*n* = 1076 USA	≥3 atypical nevi >50 nevi personal history of melanoma positive family history of melanoma extensive lentiginosis	approx. 27 regional photographs on standard poses; additional photographs were taken to monitor atypical lesions in other regions FinePix S2 Pro digital camera dermoscopy allowed, no SDDI	baseline TBP
Kelly 1997	prospective single cohort	>18 years mean age 37 years (range 18–67) *n* = 278 (female *n* = 162, male *n* = 116) Australia	dysplastic melanocytic nevi	systematic set of 14 baseline photographs with definition of standard views epiluminescence microscopy	baseline TBP
Moloney 2014	prospective single cohort	>18 years median age 53 years (range 21–85) *n* = 311 Australia	4 groups: (i) personal history of at least one invasive melanoma and dysplastic nevus syndrome (ii) personal history of at least one invasive melanoma and a family history of at least three first-degree or second-degree relatives with a confirmed history of malignant melanoma (iii) personal history of at least two primary invasive melanomas with at least one occurring in the 10 years prior to recruitment for patients with only two melanomas (iv) confirmed CDKN2A (OMIM 600160) or CDK4 (OMIM123829) gene mutation	12–24 digital high-resolution photographs according to a standardized protocol SDDI allowed	serial TBP
Nathansohn 2007	prospective single cohort	0–82 years median age 34 years *n* = 895 (female *n* = 401, male *n* = 494) Israel	>20 nevi or multiple freckles multiple dysplastic nevi positive family history of melanoma children with congenital nevi	12 MP digital single-lens reflex camera and 7 MP digital camera with polarized light dermoscope standard set of 20 photos and photos of marked lesions	serial TBP
Salerni 2012	prospective single cohort	mean age 37 years *n* = 618 (female *n* = 337, male *n* = 281) Europe	moderate to severe atypical mole syndrome (>100 nevi and/or >10 atypical and/or dysplastic nevi) personal and/or family history of melanoma carriers of melanoma gene mutations other cancer risk conditions (congenital nevus, immunosuppression, genodermatosis)	standardized digital system for images (standardized method for total body mapping) total-body mapping with digital images SDDI allowed	serial TBP
**Retrospective studies**					
Drugge 2019	retrospective two cohorts (TBP ^1^ vs. non-TBP)	>18 years median age 54 years (IQR 29) *n* = 4692 (2473 with TBP) USA	no special risk factors as inclusion criteria	simultaneous image capture of 25 cameras dermoscopy allowed	serial TBP
Drugge 2020	retrospective single cohort	*n* = 218 (subgroup of Drugge 2019) USA	personal or family history of melanoma ≥4 dysplastic nevi ≥100 nevi	simultaneous image capture of 25 cameras dermoscopy allowed	serial TBP
Feit 2004	retrospective single cohort	*n* = 576 USA	no special risk factors as inclusion criteria	standardized series of poses dermoscopy allowed, no SDDI	baseline TBP
Rademaker 2010	retrospective two cohorts	*n* = 100 patients (female *n* = 53, male *n* = 47) with melanoma (*n* = 52 invasive, *n* = 48 in situ) mean age 51 years New Zealand	patients diagnosed with melanoma or melanoma-in-situ	panoramic views, macroscopic views and dermoscopic views Nikon D50 Digital SLR camera for panoramic images Hewlett Packard Photosmart 912 or Canon Photoshot G6 1/1.8 inch 7 megapixel CCD digital camera for macro/micro images SDDI	serial TBP
Risser 2007	retrospective two cohorts (TBP vs. non-TBP)	median age 33.6 years *n* = 128 (female *n* = 71; male *n* = 57) USA	multiple atypical nevi	no dermoscopy	n.r.
Strunck 2020	retrospective two cohorts	>18 years median age 40 years (range 18–84) *n* = 1955 (female *n* = 1179, male *n* = 776) US	no special risk factors as inclusion criteria	dermoscopy, but no SDDI	n.r.
Truong 2016	retrospective single cohort	>18 years median age 39.0 years *n* = 926 (female *n* = 541, male *n* = 385) USA	no special risk factors as inclusion criteria	n.r.	n.r.

^1^ Abbreviation: n.r. = not reported, SDDI = sequential digital dermoscopy imaging, TBP = total body photography.

**Table 3:** Summary of the Outcomes of Interest of the Included Studies without a Relevant Comparison of TBP vs. Non-TBP.

Study	Biopsies				Melanoma Cases				Benign-to-Malignant Ratio	Mean/Median Breslow Index
	Numbers	Due to TBP	Due to Change of Existing /Tracked Lesion	Due to Newly Emerged Lesion	Number	Due to TBP	Due to Change of Existing /Tracked Lesion	Due to Newly Emerged Lesion		
**Prospective Trials**										
Banky 2005	n.r.	n.r.	n.r.	n.r.	18 (19 per 1000 patient-year)	n.r.	n.r.	n.r.	3:1	0.39 mm
Goodson 2010	548 in 1076 patients (0.51 biopsies per patient) 273 at initial visit 275 at follow-up	168	148 of 275	20 of 275	16 at initial visit (invasive 8) 12 at follow-up (invasive 5)	n.r.	5	2	n.r.	At initial visit: median 0.39 average 0.83 mm range 0.25–>3mm At follow-up: median 0.36 mm average 0.38 mm range 0.19–0.65 mm
Kelly 1997	210	n.r.	n.r.	n.r.	20 melanomas in 16 patients (in situ 8; invasive 12)	11	n.r.	n.r.	n.r.	Median 0.40 mm
Moloney 2014	n.r.	n.r.	n.r.	n.r.	75 primary melanoma (82 absolute) 14 at baseline 61 at post-baseline	23	n.r.	n.r.	4.4:1	Median Breslow: in situ (range in situ to 0.60 mm)
Nathansohn 2007	236	n.r.	n.r.	n.r.	7	n.r.	n.r.	n.r.	n.r.	n.r.
Salerni 2012	1152 in 407 patients (mean 1.86 lesions per patient)	n.r.	n.r.	n.r.	98 in 78 patients (8.5% of excised lesions); (in situ 53)	n.r.	60	38	Monitored lesions: 11.9:1 New lesions: 8.8:1	Median 0.5 mm
**Retrospective Studies**										
Drugge 2019	268 biopsies in 218 patients	n.r.	n.r.	n.r.	65 in 218 patients (in situ 39; invasive 26) In situ/ invasive ratio 1.56:1	n.r.	n.r.	n.r.	3.1:1	n.r.
Drugge 2020	225 biopsies in 218 patients	n.r.	149 of 225	76 of 225	67 of 225 biopsies (in situ 44; invasive 23)	n.r.	n.r.	n.r.	n.r.	Median 0.30 mm (range 0.12–0.65 mm)
Feit 2004	77 melanocytic lesions (93 in total)	n.r.	47	27	27 in 12 patients (in situ 21; invasive 6)	n.r.	20	5	n.r.	Absolut values of tumors: 0.2 mm/0.25 mm/0.25 mm/0.35 mm /0.9 mm/1.1 mm

**Table 4:** Summary of the Outcomes of Interest of the Included Studies with a Comparison of TBP vs. Non-TBP.

Study	Biopsies				Melanoma Cases				Benign-to-Malignant Ratio	Mean/Median Breslow Index
	Numbers	Due to TBP	Due to Change of Existing /Tracked Lesion	Due to Newly Emerged Lesion	Number	Due to TBP	Due to Change of Existing /Tracked Lesion	Due to Newly Emerged Lesion		
Retrospective Studies										
Rademaker 2010	n.r.	n.r.	n.r.	n.r.	n.r.	n.r.	n.r.	n.r.	n.r.	Breslow index with TBP (vs. register data): <0.75 mm: 69% (vs. 52%) 0.76–1.49: 21% (vs. 22%) 1.5-3.0: 8% (vs. 15%) >3.0 mm: 2% (vs. 11%) First visit average 0.87 mm (range 0.3–3.35) Follow-up average 0.67 (range 0.22–1.60 mm
Risser 2007	Mean: 0.81 TBSE: 0.82 (51 in 29 patients) TBP: 0.8 (53 in 28 patients)	n.r.	n.r.	n.r.	TBSE: 3 in 19 patients TBP: 0 in 16 patients	n.r.	n.r.	n.r.	n.r.	Absolute: 0.3, 0.44 and 1.1 mm
Strunck 2020	n.r.	n.r.	n.r.	n.r.	121 (in situ 51, invasive 70)	54 changing; 11 new	65	12		Median depth - 0.33 mm in follow-up group (range 0.11–1.5) - 0.83 mm in control group without follow-up (range 0.11–3.3) Median depth, if TBP was used: - 0.31 mm (range 0.11–1.5) Median depth without use of TBP: - 0.48 mm (0.10–3.3)
Truong 2016	Pre-TBP 3489 biopsies in 589 patients (mean 5.92) Post-TBP 921 biopsies in 589 patients (mean 1.56)	n.r.	n.r.	n.r.	Pre-TBP: 278 biopsies with 32 melanomas (in situ 14; invasive 18) Post-TBP: 1419 biopsies with 93 melanomas (in situ 46; invasive 47)	n.r.	n.r.	n.r.	Pre-TBP 7.7 Post-TBP 14.3	Range: - Pre-TBP: 0.2–3.0 mm - Post-TBP: 0.11–2.1 mm

Abbreviation: n.r. = not reported, TBP = total body photography, TBSE = total body skin examination.

## Data Availability

The data presented in this study are available on request from the corresponding author.
